# Evaluation of Thiol Homeostasis in Multiple Sclerosis and Neuromyelitis Optica Spectrum Disorders

**DOI:** 10.3389/fneur.2021.716195

**Published:** 2021-08-30

**Authors:** Burak Arslan, Gökçe Ayhan Arslan, Aslı Tuncer, Rana Karabudak, Aylin Sepici Dinçel

**Affiliations:** ^1^Department of Medical Biochemistry, Gazi University Faculty of Medicine, Ankara, Turkey; ^2^Department of Medical Biochemistry, Erciş State Hospital, Van, Turkey; ^3^Department of Neurology, Erciş State Hospital, Van, Turkey; ^4^Department of Neurology, Hacettepe University Faculty of Medicine, Ankara, Turkey

**Keywords:** multiple sclerosis, neuromyelitis optica spectrum disease, myelin oligodendrocyte glycoprotein, thiol, oxidative stress

## Abstract

**Objectives:** The aim of this pilot study was to evaluate dynamic thiol-disulfide homeostasis as a novel oxidative stress parameter in multiple sclerosis (MS), neuromyelitis optica spectrum disorders (NMOSD), and myelin oligodendrocyte glycoprotein antibody-associated disease (MOGAD) to better understand the role of thiol homeostasis in neuroimmunological diseases.

**Methods:** A total of 85 participants were included in this study, consisting of 18 healthy controls, 52 patients diagnosed with MS, seven with NMOSD, and eight with MOGAD. We measured total thiol (–SH+-S–S–) and native thiol (–SH) levels in the serum of all the participants, and in a subset of patients (*n* = 11), these parameters were investigated in paired cerebrospinal fluid (CSF) and serum samples. Dynamic disulfide concentrations were calculated separately. Finally, we determined if there was any relationship between clinical features and dynamic thiol homeostasis.

**Results:** There was a statistically significant difference between serum and CSF levels of biomarkers of thiol homeostasis. Serum total thiol (317.88 ± 66.04) and native thiol (211.61 ± 44.15) levels were significantly lower in relapsed patients compared to those in remission (368.84 ± 150.36 vs. 222.52 ± 70.59, respectively).

**Conclusions:** Oxidative stress plays a crucial role in the physiopathology of neuroimmunological diseases. Thiol homeostasis may be useful for monitoring disease activity.

## Introduction

Multiple sclerosis (MS) is a chronic autoimmune demyelinating disease of the central nervous system (CNS) that causes neuronal damage which underlies many of the clinical features. It was first described by Jean-Martin Charcot in 1868 and is the most common cause of physical and cognitive disability in young adults (1). It has been estimated that ~2.3 million people worldwide suffer from MS (2). The disease is thought to occur in genetically susceptible individuals as a result of environmental factors and potential triggers such as Epstein Barr virus seropositivity, obesity, smoking, and Vitamin D deficiency. However, the exact cause of MS remains unknown (3–7). Myelin sheaths, oligodendrocytes, and inevitably, axons and neurons are damaged. MS is characterized by the presence of demyelinated areas called plaques in the brain and spinal cord. In early stages of the disease, the permeability of the blood–brain barrier increases due to the migration of peripheral immune cells to the central nervous system, and a significant inflammation of the parenchyma is observed (8). Importantly, both neuroinflammation and oxidative stress, which are closely interrelated, play a crucial role in the pathophysiology of MS (9–11). MS can be diagnosed based on various clinical presentations ranging from motor and autonomic dysfunction to psychobehavioral deficits (12–14). Although the presentation of the disease is generally relapsing–remitting (85%), ~15% of it is progressive from the onset. Secondary progression occurs in the later stages of the disease in most patients with relapsing–remitting MS (9).

Neuromyelitis optica spectrum disorder (NMOSD) is a type of astrocytopathy in which aquaporine-4 (AQP4)-IgG antibodies bind to AQP4 water channels on the end feet of astrocytes, leading to immune-mediated inflammation and secondary demyelination that can occur in varying degrees (15, 16). This disease is typically characterized by clinically moderate to severe attacks, and secondary progression is not usually seen. It is more common in middle-aged women, and complete recovery after attacks is usually rare (17, 18).

Myelin oligodendrocyte glycoprotein (MOG)-Ab-associated disease (MOGAD) has been proposed to be distinctly different from seronegative NMOSD. In this context, anti-MOG antibodies are developed against MOG protein expressed on myelin sheath and oligodendrocytes (19). Although MOGAD can occur at any age, younger adults are more susceptible. The course of the disease is characterized by monophasic or relapsing features. Currently, the relationship of MOGAD with MS and NMOSD is being discussed. Although this disease has clinical and radiological similarities with NMOSD and MS, the current trend is to classify MOGAD as a different entity from MS and NMOSD (20–23).

Multiple sclerosis treatment has emerged in the last 15 years. Today, there are different choices of disease-modifying treatments for different phases of the disease and medications with different immune actions. Glatiramer acetate and interferon beta 1a, teriflunomide, and dimethyl fumarate are the first-line drugs, whereas there are anti-CD20 treatment options as ocrelizumab and barrier blockers as natalizumab and fingolimod. For neuromyelitis optica, there are guidelines for using rituximab, azathioprine, mycophenolate mofetil, and eculizumab (15, 24).

Autoimmune mediated inflammatory tissue damage increases the production of reactive oxygen molecules that cause cell damage ultimately leading to cell death. In order to maintain the balance at the cellular level, the antioxidant system is activated in response to the production of reactive oxygen molecules (25). If the balance between these systems is not maintained, oxidative stress can emerge at the cellular level. There are many antioxidant defense mechanisms at the cellular level to eliminate oxidative stress, such as the glutathione system, catalase, thioredoxin-peroxiredoxin, alpha-ketoglutarate dehydrogenases, and endogen and exogen antioxidant molecules (26, 27). However, the measurement of only one of these does not give us adequate information about oxidative balance. Thiol-disulfide homeostasis is one of the most common parameters used to evaluate the redox balance in organisms. Thiols are organic complexes that contain a sulfur and a hydrogen group attached to a carbon. Reactive oxygen species (ROS) transfer their excess electrons to thiols, thus oxidizing them. As a result of this process, disulfide bonds are formed. These disulfide bonds are then broken down depending on the oxidant-antioxidant status of organism. Thiols are considered to be antioxidant molecules due to their reducing properties. This chain of events is termed *dynamic thiol-disulfide homeostasis* (28–31). Therefore, by evaluating this dynamic homeostasis, we can determine the oxidative status of an individual without having to measure oxidant and antioxidant molecules separately with a single run.

Neuroinflammation is one of the most common pathways seen in diseases associated with neurodegeneration (32). Importantly, certain diseases, particularly those characterized by neuroinflammation of the CNS, can lead to increased risk of oxidative stress and high oxygen consumption in the CNS. Thiol-disulfide homeostasis has been shown to play a role in the pathogenesis of many diseases, including cardiovascular diseases, rheumatoid arthritis, Parkinson, Alzheimer's, amyotrophic lateral sclerosis, and MS (33–38).

Previous studies have reported on the association between MS and oxidative stress (39, 40). Moreover, many studies using blood, cerebrospinal fluid, and post-mortem brain samples from patients with MS have shown impairments in reduction-oxidation (redox) homeostasis (26). However, no study to date has assessed MS, NMOSD, and MOGAD and their relationship with thiol-homeostasis. Although there have been hypotheses about the pathophysiology of the inflammatory CNS diseases that can mimic MS in the last decade, there are still many gaps that need to be filled. Although the relationship between inflammation and oxidative stress has been demonstrated in the pathophysiology of MS, this relationship is not yet clear in diseases such as NMOSD and MOGAD (15). In this study, we tried to fill these gaps in the disorders.

Distinguishing MS from CNS diseases that can mimic MS is important in terms of therapeutic and prognostic approach. In this context, thiol-disulfide homeostasis would not only provide information about the oxidative status but perhaps distinguish these diseases from each other.

In this study, we aimed to evaluate dynamic thiol-disulfide homeostasis in different subgroups of MS, NMOSD, and MOGAD patients along with their clinical features compared to healthy controls (HCs).

## Methods

### Subjects

In total, seven patients with AQP4-Abs + NMOSD (seven serum samples), eight patients with MOG-Abs + MOGAD (eight serum samples), and 52 patients with seronegative MS (52 serum, 11 CSF samples, and 11 paired samples) were enrolled in the study. The MS group consisted of three subgroups: (1) relapsing-remitting MS (RRMS), *n* = 31; (2) secondary progressive MS (SPMS), *n* = 15; and (3) primary progressive MS (PPMS), *n* = 6. All patients with CSF samples were newly diagnosed by neurologists (RRMS = 11 CSF samples).

Serum samples were also collected from 18 HCs (18 serum samples) who were sex- and age-matched to the patient cohort. Both AQP4-Abs and MOG-Abs were verified twice (>1:40 titer) by a commercially available fixed cell-based assay (Euroimmun, Lubeck, Germany). Serum and CSF samples were collected at the Department of Neurology, Hacettepe University (Ankara, Turkey) and then stored at −80°C in the Department of Medical Biochemistry, Gazi University (Ankara, Turkey). Additional CSF samples were gathered within patients undergoing relapse. All samples were collected between January 1, 2019, and January 1, 2020 with a maximum of 2 months between the symptom onset of relapse and sample collection. The treatment-naive group consisted of patients (*n* = 30) who had not received steroid treatment in the last month and who had not been treated with immunomodulatory therapy in the last 3 months before sample collection. Our cohort also included patients who received different types of immune modulator therapy (*n* = 37) in addition to the treatment-naive group (all of the treatment-naive patients were in MS subtypes).

### Diagnosis, Clinical Evaluation, and Data Collection

AQP4-Abs-positive NMOSD and MOG-Abs-positive MOGAD patients were diagnosed according to previously determined consensus criteria (18). MS diagnosis was made according to the modified McDonald criteria (9). Medical records, laboratory data, and magnetic resonance imaging (MRI) findings of the patients were reviewed retrospectively. We carried out clinical evaluations using the neurological examination and Expanded Disability Status Scale (EDSS). Relapse was defined as a monophasic clinical episode with patient-reported symptoms and objective findings typical of MS, reflecting a focal or multifocal inflammatory demyelinating event in the CNS, developing acutely or subacutely, with a duration of at least 24 h, with or without recovery, and in the absence of a fever or infection (9).

Demographics data (sex, age, age at onset of the disease, and time of first complaints), clinical features (number of relapses, presenting symptoms, disease duration, disease subtype for MS, acute and maintenance treatments, and neurological findings at last follow-up), laboratory findings (serum and cerebrospinal fluid examinations, MOG-IgG titer, AQP4-IgG titer, and biomarker levels), and radiological findings were examined retrospectively.

### Standard Protocol Approvals, Registrations, and Patient Consents

Our study was approved and reviewed by the ethics committee of the faculty of Medicine, at Gazi University (ethical approval number: 33-14.01.2019). All individuals provided written informed consent.

### Biomarker Analysis

CSF and serum samples were aliquoted into Eppendorf tubes and stored prior to conducting the biomarker studies which were performed at different times. Centrifuge protocols were used for CSF and blood sample biobanking as previously described (41). Briefly, CSF samples were centrifuged at 400 × g for 10 min and blood samples at 2,000 × g for 10 min at room temperature.

### Cell-Based Assays

The MOG-IgG test was performed using a Euroimmun kit that utilizes a cell-based assay (CBA) employing formalin-fixed HEK293 cells transfected with full-length human MOG (reactivity at a dilution of 1:10 is positive). A baseline AQP4-IgG test was performed by a fixed-cell CBA (Euroimmun, titer >1:10 is positive) in all patients.

### Evaluation of Thiol-Disulfide Homeostasis

Serum thiol-disulfide homeostasis was determined using a novel spectrophotometric method as previously described (29). Briefly, dynamic and reducible disulfide bonds (–S–S) in the samples were reduced to free functional thiol groups (–SH) using sodium borohydride (NaBH_4_). In order to avoid the reduction of unused reduced sNaBH_4_ to dithionite-2 nitrobenzoic (DTNB), NaBH_4_ was removed with formaldehyde. Then, the native thiol (NTL) and total thiol (TTL) levels were determined after reacting with DTNB. Half of the difference of the result obtained by the subtraction of NTL amount from TTL content indicated the disulfide (DS) level. Finally, the NTL/TTL (–SH/–SH+-S–S), disulfide/NTL (–S–S/–SH), and disulfide/TTL (–S–S/–SH+-S–S) ratios were calculated.

### Statistics

All data were analyzed using the SPSS package program (ver. 21.0; IBM Corp., Armonk, NY, USA) with a 95% confidence level. Categorical variables are represented as frequency (n) and percentage (%), while numerical variables are represented as mean, standard deviation (SD), and median (M). The normality of the distribution of numerical variables was examined using Shapiro–Wilk tests. Ordinal variables were described by median and interquartile ranges (IQRs), mean and standard deviations (SD) in Gaussian distributed data, and categorical variables by counts and percentages. Spearman correlation coefficients were calculated to determine the relationship between serum and CSF biomarker levels and numeric clinical variables across all the patients and within each disease. The Mann Whitney test was used to compare groups in terms of a quantitative variables. The Kruskal–Wallis test was used to compare independent *k* groups (*k* > 2) in terms of quantitative variables. Demographic features of the participants at baseline were compared using the Fisher exact test or the Wilcoxon test. The GraphPad Prism software was used for some graphical demonstrations. Variables with two-tailed *p* < 0.05 were considered significant.

## Results

The demographic and clinical characteristics of the patients are summarized in Table 1.

**Table 1:** Demographic and clinical characteristics of the patients.

	**MS (** ***n*** **=** **52)**					
		**Progressive disease (** ***n*** **=** **21)**		**NMOSD (*n* = 7)**	**MOGAD (*n* = 8)**	**HC (*n* = 18)**
	**RRMS(*n* = 31)**	**PPMS (*n* = 6)**	**SPMS (*n* = 15)**	**AQP4+**	**MOG+**	**–**
**Gender**, ***n*****(%)**						
Female	23 (74.2)	2 (33.3)	10 (66.7)	7 (100)	3 (37.5)	13 (72.2)
Male	8 (25.8)	4 (66.7)	5 (33.3)		5 (62.5)	5 (27.8)
**Age at sampling, y**	37.2 (±11.6)	48.0 (±11.7)	45.0 (±8.07)	48.1 (±11.1)	36.8 (±7.4)	34.8 (±7.5)
**Age at onset, y**	33.7 (±12.1)	36.8 (±10.7)	29.0 (±10.6)	39.1 (±10.8)	32.1 (±10.0)	–
**Disease duration, y**	3.0 (1.0–7.0)	11 (7.2–19)	15.0 (12.0–20.0)	6.0 (2.0–15.0)	2.5 (1.2–4.7)	–
Seropositive^*^Y,N, ***n***	N, 31	N, 8	N, 16	Y, 7	Y, 8	N, 18
**Medication**, ***n*****(%)**						
Treatment Naive	24 (77.4)	4 (66.7)	2 (13.3)	–	–	–
Interferon beta 1a	3 (9.7)	–	–	–	–	–
Glatiramere acetate	4 (12.9)	–	1 (6.7)	–	–	–
Interferon beta 1b	–	–	2 (13.3)	–	–	–
Ocrelizumab	–	2 (33.3)	1 (6.7)	–	–	–
Azathioprine	–	–	3 (20.0)	3 (42.9)	2 (25.0)	–
Fingolimod	–	–	4 (26.7)	–	–	–
Natalizumab	–	–	2 (13.3)	–	–	–
Rituximab	–	–		4 (57.1)	6 (75.0)	–
**Oligoclonal band**, ***n*****(%)**						
Negative	6 (15.8)	6 (83.3)	10 (66.7)	7 (100)	5 (75.0)	–
Type 2	17 (54.8)	1 (16.7)	5 (33.3)	–	1 (12.5)	–
Type3	1 (3.2)	–	–	–	–	–
Type 2,3	1 (3.2)	–	–	–	–	–
Type 4	–	–	–	–	1 (12.5)	–
**Clinical presentation**, ***n*****(%)**						
Motor symptoms	7 (22.6)	3 (50.0)	4 (26.7)	–	–	–
Polysymptomatic presentation	8 (25.8)	3 (50.0)	4 (26.7)	–	–	–
Brainstem symptoms	1 (3.2)	–	2 (13.3)	–	–	–
Optic neuritis	4 (12.9)	–	2 (13.3)	4 (57.1)	6 (75.0)	–
Sensorial symptoms	6 (19.4)	–	3 (20.0)	–	–	–
Sphincter dysfunction	1 (3.2)	–	–	–	–	–
Cerebellar symptoms	4 (12.9)	–	–	–	–	–
Transverse myelitis	–	–	–	2 (28.6)	1 (12.5)	–
ON–TM	–	–	–	–	1 (12.5)	–
Area postrema syndrome	–	–	–	1 (14.3)	–	–
**Relapse** ^**^ **Y, N**	Y, 14 (45.2)	N, 6 (100.0)	N,15 (100.0)	Y,1 (14.3)	Y,2 (22.2)	–
**Time of complaint beginning, y**	4.0 (2.0–8.0)	12 (8.2–20.0)	16.0 (13.0–21.0)	8 (4–27)	8.5 (3.5–11.7)	–
**EDSS**	2.0 (2.0–3.2)	5.7 (4.1–6.6)	4.7 (3.8–6.6)	4 (2.5–4)	1.5 (0–3.7)	–
**Relapse number**	2.5 (1–4)	0	5.0 (3.0–15.0)	3.5 (2.7–11.25)	3.5 (1.5–5.7)	–
**GD+** **enhancing lesions**	0 (0–1)	0	0	0 (0–0.25)	1 (0–1)	–
**MRI,T2 lesions**	17.0 (8.5–40.0)	25 (25–30)	70 (25–100)	1.5 (0–2.25)	1 (0–2.25)	–

*MS, multiple sclerosis; RRMS, relapsing–remitting multiple sclerosis; PPMS, primary progressive multiple sclerosis; SPMS, secondary progressive multiple sclerosis; NMOSD, neuromyelitis optica spectrum disorders; MOGAD, myelin oligodendrocyte glycoprotein-Ab-associated disease; HC, healthy control; AQP4+, aquaporine-4 positive; MOG+, Myelin oligodendrocyte glycoprotein positive; y, year; Y, yes; N, no; EDSS, Expanded Disability Status Scale; GD, gadolinium; MR, magnetic resonance imaging; –, not available*.

* *Seropositive is indicated as Yes or No*.

** *Relapse at the time of sampling is indicated as Yes or No*.

There were significantly more females among the subgroups of diseases compared to males (*p* < 0.05), except for the PPMS (male = 66.7%) and MOGAD (male = 62.5%) groups. The SPMS group (median: 15.0, IQR: 12.0–20.0) had the highest median level of disease duration (*p* < 0.05) and the highest number of GDT2 + lesions (median: 70, IQR: 25–100) (*p* < 0.05). The PPMS group (median: 5.7, IQR: 4.1–6.6) had the highest median EDSS level (*p* < 0.05).

**Figure 1:**
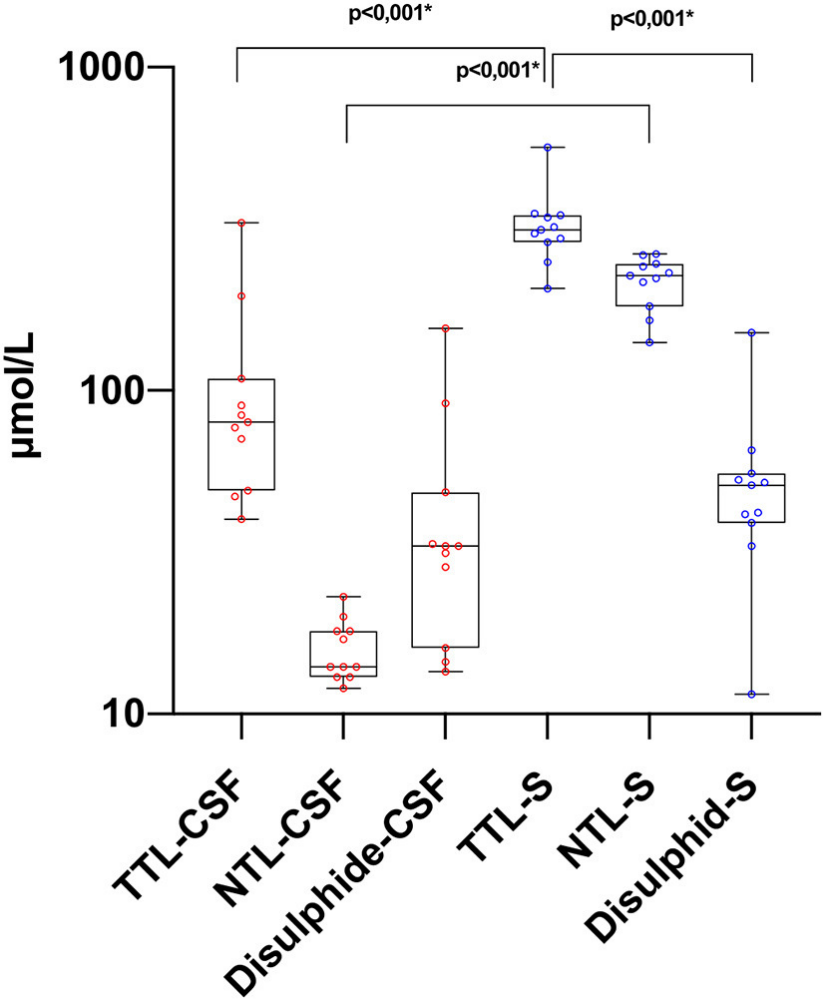
Thiol levels in paired CSF and serum samples. TTL, total thiol; NTL, native thiol; CSF, cerebrospinal fluid. ^*^The statistical significance is marked with asterisks (Mann–Whitney *U*-test).

**Figure 2:**
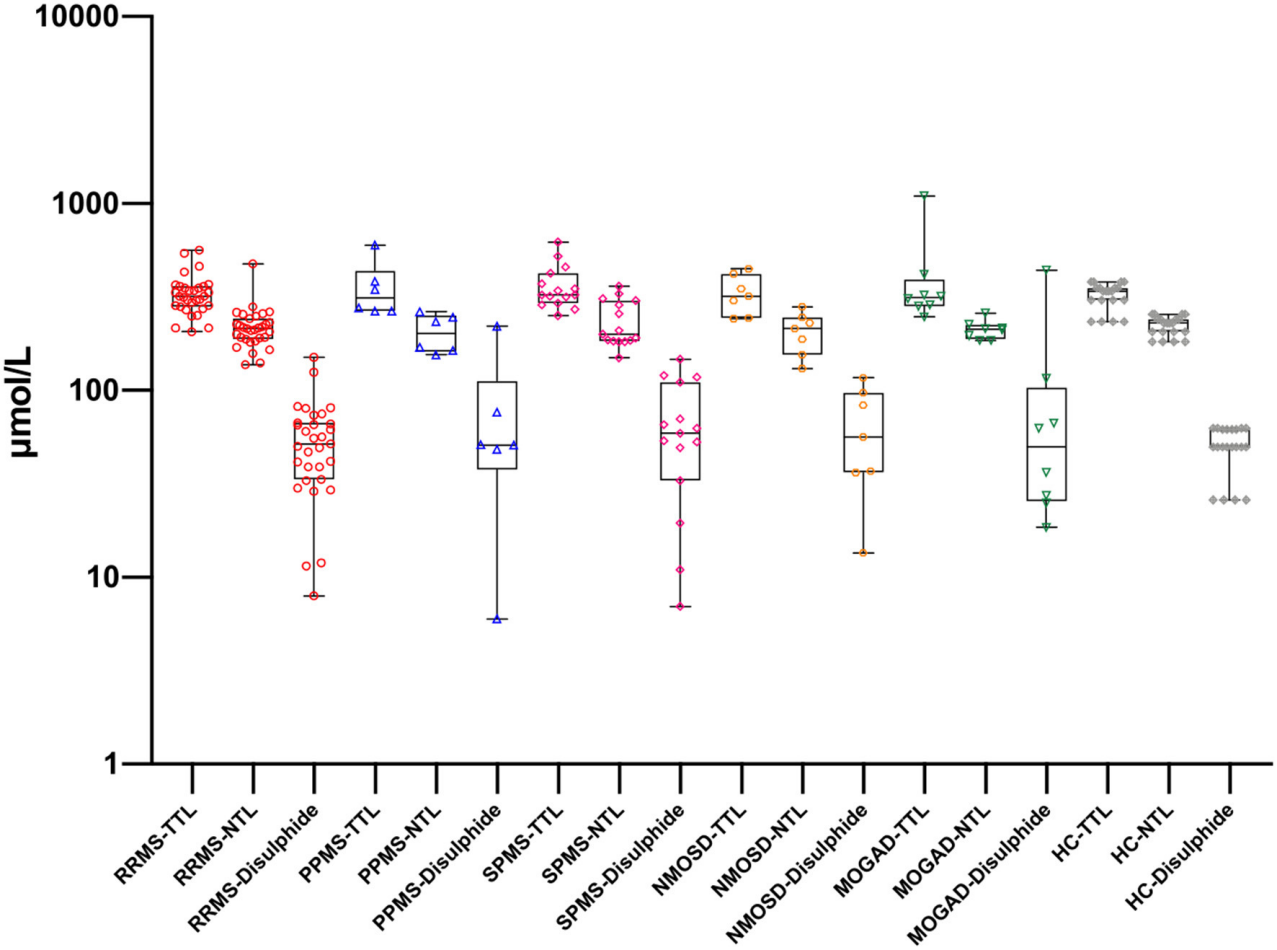
Thiol levels in serum samples with different disease subgroups and healthy controls. RRMS, relapsing-remitting multiple sclerosis; PPMS, primary progressive multiple sclerosis; SPMS, secondary progressive multiple sclerosis; NMOSD, neuromyelitis optica spectrum disorders; MOGAD, myelin oligodendrocyte glycoprotein-Ab-associated disease; HC, healthy control; TTL, total thiol; NTL, native thiol. There is no statistically significant difference for the same biomarker between groups.

There was a statistically significant difference between serum and CSF levels of biomarkers (*p* < 0.05). That is, serum levels of biomarkers were significantly higher than CSF values (*p* < 0.001; Figure 1 and Table 2). No significant relationship was found between serum and CSF values of biomarkers (*p* > 0.05). There was also no statistically significant difference in terms of serum biomarkers between subgroups (*p* > 0.05; Figure 2). MOGAD serum TTL levels (410.7 ± 281.5) and PPMS serum disulfide levels (75.8 ± 74.9) were comparable between the different disease subgroups and healthy controls (*p* > 0.05; Table 2). Serum TTL (317.88 ± 66.04) and NTL (211.61 ± 44.15) levels were significantly lower in relapsed patients (368.84 ± 150.36) compared to those in remission (222.52 ± 70.59; *p* < 0.05).

**Table 2:** Serum biomarker levels in different disease subgroups.

	**MS (** ***n*** **=** **52)**						
		**Progressive disease (** ***n*** **=** **21)**		**NMOSD (*n* = 7)**	**MOGAD (*n* = 8)**	**HC (*n* = 18)**	***p*-value** ^**a**^
	**RRMS(*n* = 31)**	**PPMS (*n* = 6)**	**SPMS (*n* = 15)**	**AQP4+**	**MOG+**		
**CSF protein**	37.57 (26.56–43.47)	47.3	27.70 (25.54–43.60)	77.42 (±35.4)	45.31 (31.91–77.56)	–	*>0*.*05*
**IgG index**	0.98 (0.73–1.19)	0.41	0.87 (0.64–1.51)	0.61 (±0.1)	0.58 (±0.09)	–	***<0***.05^*^
**Total thiol (–SH+-S–S)(μmol/L)**	337.4 (±100.0)	357.1 (±127.8)	366.0 (±101.8)	332.5 (±80.0)	410.7 (±281.5)	316.8 (±51.0)	*>0*.*05*
**Native thiol (–SH)(μmol/L)**	216.0 (±73.1)	205.5 (±47.8)	235.0 (±66.5)	206.5 (±51.9)	212.3 (±24.8)	219.3 (±25.7)	*>0*.*05*
**Dynamic disulfide (–S–S)(μmol/L)**	60.6 (±33.8)	75.8 (±74.9)	65.5 (±42.0)	63.0 (±37.3)	49.7 (25.6–103.7)	48.7 (±13.7)	*>0*.*05*
**S–S–/–SH**	0.26 (0.18–0.39)	0.43 (±0.50)	0.30 (±0.20)	0.33 (±0.22)	0.24 (0.10–0.56)	0.21 (±0.04)	*>0*.*05*
**–S–S–/(–SH+-S–S–)**	0.17 (±0.08)	0.18 (±0.11)	0.17 (±0.08)	0.18 (±0.08)	0.18 (±0.11)	0.15 (±0.02)	*>0*.*05*
**–SH/(–SH+-S–S–)**	0.64 (±0.16)	0.62 (±0.22)	0.65 (±0.16)	0.63 (±0.17)	0.63 (±0.22)	0.69 (±0.04)	*>0*.*05*

*MS, multiple sclerosis; RRMS, relapsing–remitting multiple sclerosis; PPMS, primary progressive multiple sclerosis; SPMS, secondary progressive multiple sclerosis; NMOSD, neuromyelitis optica spectrum disorders; MOGAD, myelin oligodendrocyte glycoprotein-Ab-associated disease; HC, healthy control; AQP4+, aquaporine-4 positive; MOG+, myelin oligodendrocyte glycoprotein positive; /, ratio*.

* *The statistical significance is marked with asterisk and bold*.

a *Kruskal–Wallis test*.

Serum TTL was negatively correlated with the number of relapses in relapsed patients (*p* = 0.027, *r* = −0.521). Serum NTL levels were negatively correlated with age at onset and EDSS, respectively (*p* = 0.045, *r* = −0.477; *p* = 0.031, *r* = −0.508). The age at sampling and number of GDT2 + lesions were positively correlated (*p* = 0.018, *r* = 0.537). When all patient subgroups were evaluated, there was a negative correlation between serum NTL levels and age at sampling, age at onset (*p* = 0.024, *r* = −0.244; *p* = 0.031, *r* = −0,264; respectively). For the RRMS group, there was a negative correlation between serum TTL, NTL, and EDSS (*p* = 0.017, *r* = −0.439; *p* = 0.004, *r* = −0.522; respectively). For the PPMS group, there was a positive correlation between serum disulfide and disease duration (*p* = 0.024, *r* = 0.870).

## Discussion

Previous studies have investigated the oxidative stress markers and antioxidant molecules that develop in MS patients (42–45). Furthermore, their relationships with disability and subtypes of disease have also been examined (46).

Although previous studies have assessed thiol homeostasis in MS (47, 48), to the best of our knowledge, this is the first study to evaluate it within MS subtypes (NMOSD and MOGAD) compared to HCs. Moreover, in these studies, very limited clinical information was used, and its relationship with clinical findings was largely unexplored. Interestingly, until now, no study has investigated dynamic thiol disulfide homeostasis in NMOSD and MOGAD.

We found that there was a higher ratio of women to men among the MS subgroups, except in the PPMS and NMOSD groups, which is consistent with the literature (11, 15). Furthermore, similar to previous studies, we found that the duration of the disease was longer in the progressive MS group. Likewise, the progressive group had a higher EDSS score than the RRMS group, even if there were naive patients in RRMS group.

Importantly, we are the first to evaluate CSF and serum thiol homeostasis together. The brain parenchyma produces a high rate of oxidative radicals due to its high oxygen consumption (49, 50). In our study, it was observed that TTL, NTL, and disulfide CSF levels were lower than serum values. Presumably, in this case, peripheral oxidative damage and dynamic thiol homeostasis that emerges in response also plays a role. These findings suggest that CSF TTL, NTL, and disulfide levels may be used to evaluate disease status in patients who undergo CSF sampling for diagnostic purposes.

When oxidative stress increases, thiol groups of proteins react with oxidants and turn into disulfide bonds. These formed bonds can then be reduced to thiol groups again, reestablishing thiol-disulfide homeostasis (51).

In our study, the SPMS group had the highest serum mean TTL levels among the MS group. The SPMS group also had the highest number of relapses and longest disease duration. This suggests that as new disulfide bonds are formed, more thiol groups can be reduced in this group. The MOGAD group had the highest TTL levels among the disease subtypes. This may actually be related to the pathophysiology of this disorder (15), as the main problem in this disease is the MOG-IgG Ab that develops against myelin sheath, which causes oxidative damage, thereby leading to an increase in TTL levels.

The PPMS group had the highest serum disulfide levels among the disease subtypes. This condition, which is indicative of oxidative damage, may also be associated with neuronal damage. In addition, the negative correlation between serum TTL, NTL, and EDSS in the RRMS group suggests that these values could be used in the assessment of disability.

Plasma membrane is like the primary sensor of the cells' extrinsic stressors. Excess amounts of ROS cause cell damage and death by disrupting plasma membrane functions. These mechanisms are believed to play a major role in the pathophysiology of many neurodegenerative diseases. However, low amounts of membrane-associated ROS may activate adaptive pathways against stress. There is increasing evidence for the hormetic role of membranes in states of low and transient oxidative stress. Under normal conditions, membrane lipid peroxidation is usually low. However, an increase in oxidative stressors in cells such as neurons with high energy demand and oxygen consumption may deteriorate the hormetic roles of the membrane (52–55). Perhaps, the low thiol levels in relapsed patients in our study can be explained by the deterioration in the hormetic role of the membrane. In addition, irrespective of the cause, the increase in cellular oxidative stress at subtoxic levels causes a neuroprotective effect known as “preconditioning.” Preconditioning signal provides cellular protection through hormesis, which is a dose-response phenomenon characterized by a high-dose inhibition and low-dose stimulation (56–59). Thanks to this chain of events, neuroprotection is also provided.

Protein thiols are important mediators of multiple metabolic, signaling, and transcriptional processes; they play a key role in the regulation of redox sensing and cellular redox status. “Vitagenes,” which play a key role in oxidative stress protection, are protective genes that control pro-survival pathways that must be activated in response to cellular stress. They encode cytoprotective heat shock proteins, thioredoxin, and sirtuins (60). Under normal physiological conditions, long-term health is maintained by protein homeostasis. When this homeostasis is disrupted, cellular stress is released and heat shock proteins, including chaperones, are produced and contribute to cell survival (61, 62). In this study, the SPMS group had the highest serum mean TTL levels among the MS groups. According to this result, we may assume that vitagen activity may have increased, and pro-survival mechanisms are activated for the continuity of protein homeostasis.

Our study had some limitations. Particularly, the number of patients in the NMOSD and MOGAD groups was lower than the other groups. While the numbers in this group reflected all of the patients in our tertiary center, all of them were being treated with immunomodulatory therapy. In addition, due to ethical considerations, we could not obtain CSF samples from HCs; thus, there were no CSF samples included in our HC group. Furthermore, the number of patients in our progressive MS group was not close to that of the RRMS group, either. In addition, our patient population did not include more rare MS subtypes, such as clinically isolated syndrome (CIS) and radiologically isolated syndrome (RIS). Although this study demonstrates the usefulness of assessing dynamic thiol homeostasis among neuroinflammatory diseases of the CNS, future studies incorporating larger patient cohorts are needed.

## Conclusions

Oxidative stress plays a crucial role in the physiopathology of neuroimmunological diseases. This study is the first to assess thiol homeostasis among different subgroups of MS and NMOSD. Previously, thiol homeostasis has only been evaluated using serum. In future studies, CSF and serum values of thiols should be examined in detail using a larger number of paired serum and CSF samples. Perhaps in the near future, thiol status can be used by itself or in combination with other candidate biomarkers to monitor oxidative status and disease activity.

## Data Availability Statement

The original contributions presented in the study are included in the article/supplementary material, further inquiries can be directed to the corresponding author/s.

## Ethics Statement

The study was approved and reviewed by the ethics committee of the faculty of Medicine, Gazi University. The number of the ethical approval is 33-14.01.2019. The patients/participants provided their written informed consent to participate in this study.

## Author Contributions

All authors listed have made a substantial, direct and intellectual contribution to the work, and approved it for publication.

## Conflict of Interest

The authors declare that the research was conducted in the absence of any commercial or financial relationships that could be construed as a potential conflict of interest.

## Publisher's Note

All claims expressed in this article are solely those of the authors and do not necessarily represent those of their affiliated organizations, or those of the publisher, the editors and the reviewers. Any product that may be evaluated in this article, or claim that may be made by its manufacturer, is not guaranteed or endorsed by the publisher.
